# A review and revisit of nanoparticles for antimicrobial drug delivery

**DOI:** 10.25122/jml-2021-0097

**Published:** 2022-03

**Authors:** Kai Bin Liew, Ashok Kumar Janakiraman, Ramkanth Sundarapandian, Syed Haroon Khalid, Fizza Abdul Razzaq, Long Chiau Ming, Abdullah Khan, Anandarajagopal Kalusalingam, Pit Wei Ng

**Affiliations:** 1. Department of Pharmaceutical Technology and Industry, Faculty of Pharmacy, University of Cyberjaya, Cyberjaya, Selangor, Malaysia; 2. Department of Pharmaceutical Technology, Faculty of Pharmaceutical Sciences, UCSI University, Kuala Lumpur, Malaysia; 3. Karpagam College of Pharmacy, Othakalmandapam, Tamil Nadu, India; 4. Faculty of Pharmaceutical Sciences, Government College University, Faisalabad, Pakistan; 5. PAPRSB Institute of Health Sciences, Universiti Brunei Darussalam, Gadong, Brunei Darussalam; 6. School of Pharmacy, KPJ Healthcare University College, Nilai, Negeri Sembilan, Malaysia; 7. Department of Pharmacy, National University Hospital, Singapore

**Keywords:** nanomedicine, nanoparticle, anti-infective agents, antibiotic, safety

## Abstract

Antimicrobials are widely used to treat bacteria, viruses, fungi, and protozoa. Therefore, research and development of newer types of antimicrobials are important. Antimicrobial resistance has emerged as a major challenge to the healthcare system, although various alternative antimicrobials have been proposed. However, none of these show consistent and comparable efficacy to antimicrobials in clinical trials. More recently, nanoparticles have emerged as a potential solution to antimicrobial agents to overcome antimicrobial resistance. This article revisits and updates applications of various types of nanoparticles for the delivery of antimicrobial agents and their characterization. Though nanoparticle technology has some limitations, it provides an innovative approach to pharmaceutical technology. Furthermore, nanoparticles offer a variety of advantages, such as enhancement of solubility and permeation, leading to better efficacy. In this article, approaches commonly employed to improve antimicrobial therapy are discussed. These approaches have advantages and applications and provide a broader opportunity for pharmaceutical scientists to choose the proper method per the desired outcome.

## INTRODUCTION

In the era of COVID-19, antimicrobial resistance has become the biggest challenge because of the sharp increase in hospitalization and empirical broad-spectrum antimicrobial use. Antimicrobial-resistant bacteria are a cause of widespread inappropriate, over-prescribing of antimicrobials. More importantly, antimicrobials are also heavily misused in the agricultural field, especially livestock. This leads to indirect consumption of antimicrobials and resistant bacteria, which exist in the food chain of humans. It must be noted that the knowledge regarding antimicrobial resistance among healthcare professionals or students still has plenty of room for improvement [1–3]. The consequences of uncontrolled antimicrobial resistance pose a tremendous threat to clinical outcomes and increase mortality and healthcare costs [4]. It was estimated that the health care cost associated with the treatment of infectious diseases in the United States (US) is approximately 120 billion dollars annually [5]. More than two million antimicrobial-resistant infections occur annually in the US, leading to 23,000 deaths [6]. On the same note, in the European Union, antimicrobial-resistant infections contributed to approximately 25,000 deaths annually [7]. Some examples of antimicrobial resistance microbes are methicillin-resistant *Staphylococcus aureus* (MRSA), drug-resistant *Streptococcus pneumoniae* and vancomycin-resistant *Enterococcus faecium* (VRE), multiple drug-resistant *Tuberculosis mycobacterium* and *Neisseria gonorrhoeae* [8–10].

In a recent study, more than half of the tested pathogenic bacteria are resistant to more than one commonly prescribed antibiotic or antifungal agent [11]. Although commonly, resistance will be developed over time, excessive antimicrobial use has expedited the process. To a large extent, the ability of multidrug-resistant bacteria to form a bacterial biofilm worsens the situation because this biofilm acts as a shield on human tissues and medical equipment. This is especially risky if the infected medical devices come into direct contact with the inner part of the human body [12]. The formation of biofilm has become one of the major factors that even bacteria without a known genetic basis can develop resistance and show significantly lower susceptibility to antimicrobials [13, 14]. The formation of biofilm has led to the failure of antimicrobial treatment. Biofilm matrix restricts the penetration of antimicrobial cells to the core of the bacterial cells. As a result, even prolonged infusion of antimicrobial agents will only have a little therapeutic effect on the infection. Biofilm bacteria can be 1000 times more tolerant to antimicrobial activity than planktonic cells of the same species [15].

The development of antimicrobials incurs a high cost, and fulfilling regulatory requirements is very challenging. As a result, most pharmaceutical companies do not prefer to invest in the research and development of antimicrobials [16]. To overcome the ascending trend of multi-drug resistant bacteria infection, novel bactericidal agents are needed. An alternative to the existing antimicrobial approach to treat infection is highly desirous [17]. There are many proposed alternatives to replace antimicrobials such as using plant extract with antimicrobial activities, bacteriophage therapy, predatory bacteria, and competitive exclusion of pathogens [18–20]. Unfortunately, none of these methods show consistent and comparable efficacy to antimicrobial treatment in clinical trials. A nanoparticle is one of the promising approaches that have proven its efficacy and potential in antimicrobial therapy.

### Nanotechnology-based approach

The existing limitation of conventional antibacterial therapy can be resolved to a certain degree by the application of nanotechnology. Nanotechnology has been applied in antimicrobial therapy for preventive, diagnostic, drug carriers, and synergistic functions [17]. The nanoparticle of bactericidal agents added advantages over the free form of the drug due to better solubility, enhanced penetration ability, and higher specificity. The formulator has better control on targeted drug delivery of bactericidal products including environmentally responsive, specific ligand-targeting design and combinatory delivery systems [21].

### Nanoparticles

Nanoparticles are generally particles with a size between 1–100 nm (ISO/TS 27687:2008 Nanotechnologies – Terminology and definitions for nano-objects – nanoparticle, nanofiber, and nanoplate) [22]. Nanomedicine has taken advantage of nanotechnology, often described as technologies that produced particles under 1000 nm, in the health sciences. Nanoparticles with sizes up to several hundred nm and with therapeutic activity are considered nano-medical agents. Recently, regulatory bodies such as the United States Food and Drug Administration (FDA) defined nanomaterials as a material, or end product developed to have at least one nanoscale (approximately 1 nm to 100 nm) external or internal dimension, or surface structure. It is also sometimes defined as a material or end product designed to exhibit properties or phenomena that are due to its dimension(s), including physicochemical properties or biological effects, even if these dimensions fall outside the nanoscale range, up to one micrometer (1,000 nm) [23]. The European Medicines Agency (EMA) defines nanomedicine as an application of nanotechnology in medical diagnosis or treatment, mitigating, or avoiding diseases. It has the advantage of enhancing and sometimes even imparting novel physical, chemical, and biological properties of nanometer-scale materials. Therefore, nanomedicines are engineered to have physicochemical and biological properties due to their size and morphology of the surface, distinguishing them from drugs of low molecular weight [24].

The application of nanotechnology has shown promising results in the therapeutic field. Some examples but not limited to these are liposomes, nanocrystals, emulsions, and iron-carbohydrate complexes. Nanomedicines have been applied in various medical fields such as cancer therapy, inflammatory diseases, infections, and anaemia [25]. Nanomedicines can be administered through various methods, including oral, topical, and parenteral. The size and surface morphology of nanomedicines contribute to their distinct physicochemical and biological properties compared to low molecular weight drugs [26].

### Advantages of nanoparticles

#### Improved drug solubility

Nanotechnology has been applied to improve the solubility of poorly aqueous soluble drugs. Owing to solubility problems, low permeability, limited bioavailability, and other poor biopharmaceutical properties, many drugs with promising therapeutic activity failed at the clinical level [27, 28].

#### Enhanced stability

With proper selection of the surfactants and stabilizers, nanosuspensions can produce a physically stable alternative form of the product. Nanoparticles have offered better stability against coalescence. Moreover, nanoparticles possess an interior core that allows the assimilation of hydrophobic drugs, resulting in enhanced stability. Studies have reported that nano-formulations can improve the stability of protein drugs through PEGylation. Solid lipid nanoparticles can be used for both hydrophilic and hydrophobic drug delivery [29].

#### Reduced side effects

Nanomedicine is often more specific in delivering drugs to a targeted site. As a result, the side effects can be minimized. Polymers are often used to encapsulate drugs in nanoparticles and enhance the specificity of drugs to targeted receptors, thereby enhancing the therapeutic effectiveness of the drug and mitigating the side effects [30].

#### Multimodal approach of anti-bacterial activities

A nanoparticle consists of a few components that demonstrate antimicrobial activities. An example was presented by Lam *et al.* [31] in which the nanoparticle of an antibiotic encapsulated in an anti-bacterial core material (metal or metal oxide) surrounded with a polymeric shell exhibits antibacterial activity; with this combinational strategy, the nanoparticle complex can deliver three different types of anti-bacterial mechanisms which increases the chance of therapeutic effectiveness. Wu *et al.* [32] developed zinc-doped copper oxide prickly nanoparticles demonstrating high bactericidal activity.

### Brief review on various applications of nanoparticles

There is a lot of research on nanoparticles being used in various fields. A summary of the review on various applications of nanoparticles is presented in the Supplementary Table.

Supplemental data file. Supplementary Table. References and characteristics of various nanoparticle applications.

#### Nanoparticles as antibacterial agents

##### Lipid-based nanoparticles

Lipid-based nanoparticles have been widely studied and developed to be used in the antimicrobial field. Liposomes, nanostructured lipid carriers (NLC), and solid lipid nanoparticles (SLN) are the lipid-based nanoparticles used to effectively deliver various drugs. Liposomes are spherical lipid vesicles with a bi-layered membrane structure consisting of amphiphilic lipid molecules. The surface of the liposome is hydrophobic, while the inner core is hydrophilic. Therefore, liposomes have the advantage of delivering both hydrophilic and lipophilic drugs by incorporating them into their inner aqueous core and lipid bilayer membrane structure, respectively. Furthermore, liposome delivery enhances the stability of the bactericidal agent by protecting against degradation. The surface structure of the liposome has a structural similarity with the surface of the cell membrane of bacteria. Interaction between both surfaces is possible through adsorption, endocytosis, lipid exchange, and the liposomes' fusion with the bacterial cell membrane. Fusogenic liposomes have attracted researchers' attention as the efficiency in delivering bactericidal agents is enhanced by the ability of liposomes to fuse with the bacterial cell membrane. Fusogenic liposomes have a combinational mechanism that destroys the bacterial membrane, releases their therapeutic content, and kills the bacterial cells [33].

SLNs consist of a surfactant-stabilized rigid lipid core and are moderately amorphous structures in which bilayers are not differentiated. SLNs are suitable to deliver lipophilic drugs due to the nature of their lipid particle core. The stability of the drug is enhanced, and SLNs can be scaled up for industrial mass-production [17]. However, SLN has limited application for the hydrophilic drug.

NLC has been developed to overcome the limitation of SLN related to the hydrophilic drug. Liquid lipids are used in the NLC system to stabilize the structure, allowing a biphasic drug release profile with an initial burst release followed by the sustained release of the drug [34].

Improving the antibiotic efficacy and the protection against enzymatic deactivation and fusogenicity is a specific advantage of liposomal antimicrobial nanocarriers. To avoid recurrence of infection, deep killing is required, one of the problematic features of clinical infection care. However, it is also worth investigating whether liposomal antimicrobial nanocarriers can be built to help kill bacteria that are impenetrable to many antimicrobials seeking shelter in mammalian cells.

##### Polymeric nanoparticles

Extensive research has been conducted on antibiotic conjugated polymeric nanoparticles for multidrug-resistant microbes [35]. Biocompatible and biodegradation natural and synthetic polymers have been used to form polymeric nanoparticles, which serve as a carrier for antibiotics. Bactericidal agents have been encapsulated in the internal part of a polymeric core or attached at the surface of a polymer through covalent and non-covalent bonds. Besides providing steric hindrance as protection for the drug, these polymeric nanoparticles are usually modified for stimuli triggered release of the drug [36]. Polymeric nanoparticles deliver the antibiotic to bacterial cells through passive and active targeting. Passive targeting happens when the polymer interacts with the cell membranes and disrupts the structure of the bacterial membrane making it more porous. Active targeting happens when the polymer interacts with a specific antibody and aptamer bacteriophage protein, and the drug is transported into the bacterial cells [37].

New alternatives to traditional antibiotics are antimicrobial polymeric nanoparticles, in which the nanoparticles possess intrinsic bactericidal properties. Pharmaceutical scientists can modify the charge density of antimicrobial polymer nanoparticles by optimizing antimicrobial activity and biocompatibility. Conventional antimicrobial polymer systems are modelled based on the cationic and amphiphilic properties of antimicrobial peptides (AMPs) found in a microorganism. All synthetic antimicrobial polymers such as linear polymer have cationic groups composed of different degrees of hydrophobicity embedded into the full polymer chain.

##### Dendrimer

**Figure 1.**
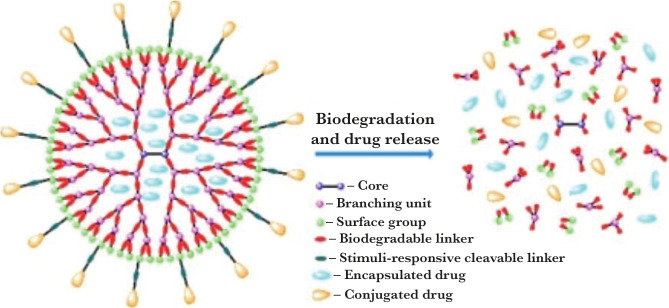
Structure of dendrimer (The image is adopted from Huang and Wu, 2018).

Dendrimers are highly branched, nanometer-scale-sized, star-shaped macromolecules, consisting of three components: the central core, the dendritic structure of the interior, and the functional surface groups of the exterior surface. The structure of the dendrimer is presented in Figure 1. Dendrimers have well-defined 3D structures with available functional groups, which ease the prediction of antibiotic binding with the dendrimer structure. Hydrophilic and lipophilic drugs can be incorporated into the dendrimer structure or attached to the surface functional groups. Dendrimers can mimic cell membranes, which makes them a suitable drug carrier. Dendrimers can enhance solubility and penetration and offer a controlled release of drugs [17]. Dendrimers can be used as anti-bacterial drug delivery systems, bacteriophobic coatings, as well as anti-bacterial agents capable of attacking bacterial cells or bacterial toxins, based on the reports summarized in this study. Dendrimers are well suited to participate in multivalent interactions because of their globular form and the location of reactive groups at the surface, allowing these compounds to interfere with the role of determinants of essential bacterial virulence. Whereas glycodendrimers provide the potential for high specificity, cationic dendrimers, either acting alone or in combination with known drugs, are promising as broad-spectrum antibiotics. Between the two methods, antimicrobial peptide dendrimers represent a middle ground. Consisting of a mixture of cationic and hydrophobic residues, due to their polypeptide nature, they act by membrane destruction but are more selective than classic cationic dendrimers. However, it should be noted that some new fields remain in this field of study, providing opportunities for further studies on the use of dendrimers as antimicrobial agents. The number and variety of chemical structures studied are small, with insufficient data available in most cases to obtain the relationships between structure and activity required to direct future developments.

##### Inorganic nanomaterials

An example of an inorganic nanomaterial used in the anti-bacterial field is mesoporous silica nanoparticles (MSN). With their mesoporous structures, MSN have some distinct advantages such as high surface area, biocompatibility, and tunable particle diameter. Modification of MSN for sustained release function has unleashed the potential of this molecule in the battle with infection. In the design of MSN, parameters controlled to enhance the functionality include surface group, size, and shape [38]. Both the planktonic bacteria and biofilms can be targeted with the modification of the surface of the functional group [39]. The applications of MSN reported in the literature include MSN for efficient antibiotic delivery [36] and hybrid anti-bacterial materials preparations by incorporating the enzymes with anti-bacterial efficiency [40], peptides [41], metallic ions/particles [42], and polymeric surface modifiers [43].

##### Metal and metal oxide nanoparticles

The use of metal nanoparticles is a common approach in the development of antimicrobial formulation. Gold and silver nanoparticles are the most researched metals in medicine and dentistry and have many possible applications. Besides gold and silver metallic nanoparticles, metal oxides of zinc, copper, and iron nanoparticles are extensively used. Various metals, as well as metal oxides NPs such as gold [44], bismuth [45], tellurium [46], copper oxide [47], zinc oxide [48], cerium oxide [49], and magnetic iron oxide [50] along with titanium magnesium and nitric oxide in contrast to their raw state, have been recognized to have elevated anti-bacterial effect. Nanoparticles do not pose any issues of microbial resistance due to numerous mechanisms of bactericidal action. Despite the toxicity caused by metals and metal oxides, they are still useful as agents to destabilize the bacterial membrane by generating ROS and eventually lead to bacteria cell death [51].

##### Cationic Polymeric nanoparticles

Nanosized particles containing Cationic polymers, either natural or synthetic, are well known for their guaranteed antibacterial activity. The sole reason behind the effective antibacterial activity of these systems is their cationic surface which can strongly interact with the surface of bacteria [52]. As far as the mechanism is concerned, different mechanisms have been described throughout the literature, including the lipophilic chains to infiltrate and rupture the membrane of bacteria. Literature enlists various polymeric NPs showing sustained bactericidal effects, superior stability, and surface binding. Some of the most prominent examples include chitosan NPs [53], lipid Nps, QA-PEI (Quaternary ammonium-Polyethyleneimine) NPs [54, 55], and peptide-based NPs [56].

##### Carbon nanostructures

Carbon nanostructures, namely fullerene [57] and carbon nanotubes [58, 59], have also been reported to exhibit antibacterial action; however, the antibacterial mechanism involved is not confirmed and needs further investigations. One of the key hurdles of this investigation revolves around dispersing these structures in water which is difficult, particularly in the case of carbon-based nanotubes [60].

##### Nanomaterials as an antibacterial agent

Nano forms of metal and metal oxide have been reported to have an enhanced antibacterial activity compared to the crude form. Nanoparticles obtained from metals and metal oxides act as anti-bacterial agents through several mechanisms. As a result, it is hard to develop resistance by microbes. Among the metal and metal oxide nanoparticles which have been studied for their antimicrobial activities are gold [61], tellurium [46], bismuth [45], copper [62] and its oxide [47], zinc oxide [48], magnesium oxide, nitric oxide [63], titanium dioxide [64], aluminium oxide [65], magnetic iron oxide [66] and cerium oxide [67].

### Approaches on the development of NP as anti-bacterial agents

#### Synthesis of nanoparticles

Various methods can be used to synthesize nanoparticles. These methods can be categorized into two major categories: the Top-Down approach and the Bottom-Up approach.

##### Top-down approach

A top-down approach is a destructive approach. The process involves breaking down a large molecule into smaller units, which are converted into NP. Mechanical milling, chemical etching, sputtering, laser ablation, and electro-explosion are a few examples of the top-down method. A physical or chemical force is applied to break down a large molecule into nano-size range particles [68]. In the literature, a top-down approach was employed to synthesize coconut shell NPs. The coconut shells were subjected to the milling process at different intervals in a planetary mill. The longer the coconut shells were subjected to milling, the smaller the coconut shell NPs particles were. The brown colour of the coconut shell faded with smaller NPs particle sizes generated as a result of milling [69]. Besides physical force, the top-down approach can also be achieved by the chemisorption method. Spherical magnetic NPs synthesis from iron oxide (Fe_2_O_3_) was generated in the presence of organic oleic acid. Continuous chemical adsorption of polyoxometalates (POM) on the carbon interfacial surface forms the basis of NPs synthesis from iron oxide (Fe_2_O_3_). Due to the adsorption, the black carbon aggregates are converted into comparatively smaller particles spherical in shape. Moreover, it may also enhance the dispersion capacity of the particles and narrow size distribution [70].

##### Bottom-up synthesis

The concept of bottom-up synthesis is the reverse of the top-down approach. This method starts with the relatively simpler substances to build up the NPs. Examples of bottom-up synthesis are biological synthesis via plants and microorganisms, spinning, template support synthesis, plasma or flame spraying synthesis, and laser pyrolysis. TiO_2_ anatase NPs composed of graphene domains were synthesized by Mogilevsky *et al.* [71]. They used titanium isopropoxide and Alizarin as the precursors in synthesizing the photoactive compound capable of photo catalytically degrading methylene blue. Via their axial hydroxyl terminal groups, Alizarin demonstrated high binding ability with TiO_2_. The size of the NPs increased with the increase in temperature. Liu *et al.* synthesized a spherical-shaped Au nanosphere with monocrystalline from an octahedral morphology via laser irradiation method [72]. The Au NPs have an average size of 75±2.6 nm diameter. More recently, the biosynthesis of NPS through plants and microorganisms has attracted the attention of many researchers. The method is considered more environmentally friendly and less toxic. Au NPs have been synthesized from the biomass of wheat and oat using microorganisms and plant extract as reducing agents [73].

### Characterization of nanoparticles

The first aspect when characterizing NPs usually constitutes the first aspect of ensuring that the produced formulations embody the required attributes and are replicable each time. The characterization of nanoparticles is a very dynamic and complicated subject as it involves plentiful parameters to be tested as well as techniques [74]. However, some of the most central attributes include the size, shape, charge, and structure of the nanoparticles [75]. In addition, the literature reveals some pointers to be kept in mind to make characterization more factual and specific.

Some of these are mentioned below:

• Take into account the type and form of the nano-formulations (powder or suspension) as it can determine the particular attributes required and can also shortlist the techniques which can be employed [76];
• If possible, employ more than one technology to characterize a single parameter;
• Compare the findings quantitatively with the standard index, and in the absence of the former index, internal referencing can also be utilized. Also, the findings collected are to be compared with reported data [77].

#### Morphological characterization

The morphology of nanoparticles is an important factor that will affect the properties of nanoparticles. The morphological analysis includes different characterization approaches, but electron microscopic approaches are always the first choice and are mostly preferred. These approaches involve scanning and transmission electron microscopy, which is labelled as the benchmark for nano morphological investigations [78]. A scanning electron microscope depicts a specimen by forcing it with a high-energy electron source that comes into contact with the specimen at the atomic level and generates (secondary) electrons, which build a high-resolution three-dimensional image of a specimen upon identification by the sensor. As far as an energy source is concerned, compared to TEM, it is lesser in strength, leading to lesser penetration and damage, and consequently, thicker samples (>100 nm) can be examined. Conductive samples can be easily imaged, but the nonconductive ones require a metallic coating [79]. TEM also works on the same principle, but it provides a higher resolution (0.05-0.1 nm), which is attributed to the strong electron source and thinner samples (<200 nm). However, the strong source does cause damage to the sample, which can be dissolved by using superior cryogenic techniques [77].

##### Particle size

Particle size is the most crucial detail of NPs. It is among the key predictors of distribution and accumulation of the NPs in subject areas inside the body. For objects of macroscopic size where size is assessed as the gap between opposite ends of an item, the nano-size entails numerous interpretations depending on the technique used to estimate it. The techniques involved in the assessment of size vary from dynamic light scattering (DLS) to microscopic methods to nanoparticle tracking analysis (NTA) [78].

Dynamic light scattering is popularly utilized in determining particle size. This technique monitors the Brownian motion of suspended nanoparticles and relates their speed to the size of nanoparticles as per the Stokes−Einstein equation and states the result as mean particle size. Although it offers an easy and quick particle size assessment, numerous findings suggest unavoidable shortcomings, especially in assessing heterogeneous samples [80]. Similarly, microscopic techniques aim to offer a precise size measurement, but they frequently involve highly complex microscope-specific sample preparatory measures that can alter particles and cause aggregation and are also unable to provide particle size distribution [81]. Another technique gaining a special place in characterizing size is nanoparticle tracking analysis. The fundamental concept involved in this technique is the Brownian motion rate to be proportional to particle size. Besides that, it is also possible to determine the size distribution portfolio with sizes varying from 10 to 1000 nm. Furthermore, this technique also exhibited admirable performance relative to dynamic light scattering and is found to be equally effective for both mono and polydisperse systems, but it is time-consuming compared to former techniques and requires the sample to be highly dense [82].

One more important consideration for determining size is the shape, as it can influence the accuracy of the size, so this assertion must be checked, especially in the case of dynamic light scattering, which presumes the particle size is spherical. Also, the category of size distribution employed should be clearly stated since it can also influence the size [83].

##### Shape

Nanoparticles are designed in different shapes, and the most commonly reported shapes include the rings, rods, tubes, spheres, fibers, and planes. The efficacy and toxicity are also attributed to the shape of nanoparticles. Studies suggest that the spherical and rod-shaped nanoparticles are comparatively safer and demonstrate higher cellular uptake [84, 85].

##### Surface charge

Each nanoparticle possesses a surface charge which can influence the particle, particularly in terms of stability. Alteration of the surface charge could significantly affect the entire stability of the particle. A standard surface charge estimation is governed by the zeta potential, which is usually defined as the potential at the rim of the outer layer. The method used to measure zeta potential is electrophoretic light scattering (ELS), which can analyze the speed of several particles concurrently. Besides that, zeta potential can also be influenced by various factors such as:

• Ionic strength of the solvent;
• Adsorption onto the surface of the particle;
• pH of the solution.

The first two factors negatively impact zeta potential leading to a decline in it, whereas the higher pH is generally responsible for the positive value and vice versa [86].

#### Structural characterization

In researching the structure and function of the bonding materials, the structural specifications are of paramount significance. They can offer detailed knowledge on the properties of the substance. Different techniques can be employed in characterizing the structure of nanoparticles. Some of the most commonly used ones are discussed in detail below [87].

XRD is one of the most powerful and flexible tools used on crystalline samples to uncover a wide array of architectural aspects [88]. The achieved data usually varies from tiny functionalities (configuration of crystal parts) to macro details (sizes and shapes) regardless of the phases. Nonetheless, the achievement of precise structural estimation might be complicated in terms of very small size (<10 nm) [89]. In addition, the XRD diffraction pattern is often impacted by amorphous character and inter variations in atomic lengths, and this could be resolved by carefully contrasting the diffraction pattern with the related nanoparticles or even their blends [90, 91].

Apart from XRD, spectroscopic techniques can also foretell composition analysis, such as IR spectroscopy, dispersive x-ray spectroscopy, and Raman spectroscopy. Among spectroscopic techniques, IR Infrared spectroscopy is the most widely used modality given its ability to identify functional groups as well as adsorbed surface molecules. Besides that, it can also note any modifications in the structure. A plethora of studies present the usefulness of IR techniques – FTIR in particular [92]. Because of its ability to determine the structure and the arrangement, Raman spectroscopy has emerged as one of the important characterization approaches for several nanoparticles. It can separate the two forms of the same nanoparticle, and it can also distinguish between phases, crystal or amorphous nature [93].

Another technique usually employed in structure characterization is known as energy dispersive x-ray spectroscopy, which is popular for its ability to provide information about the elements present in a sample and their quantity. Also, it is attached with an electron microscope, Scanning Electron Microscopy (SEM), or Transmission Electron Microscopy (TEM), which further enhances the analysis [94]. Using the SEM and TEM techniques makes it possible to detect all the morphological characteristics of the nanoparticles. With SEM, it is possible to secure a resolution down to 1.2 nm, and TEM devices could resolve images down to 0.17 nm [95].

### Information of patents

For antimicrobial therapy, metal-based nanoparticles are extensively used. Most of the patented antimicrobial nanoparticles were developed using silver metal. These products are claimed to be used to deliver drugs to treat skin conditions, including wound care, and for cosmetic purposes, to mask the bad odour due to bacterial infections and growth [96].

### Future Perspectives

Despite the significant developments in nanomedicine and its rapid growth, their progression to clinics still poses many barriers. It is also possible to see a substantial difference between the promising *in vitro* results, the very frustrating pre-clinical results, and the common effect in clinical settings. It is challenging to handle NP-host interactions. In terms of formulation, lipids in nanoparticles ensure drug encapsulation. Close examination of the cytotoxicity, inflammatory response, and immune response of the NP by performing human in vivo studies is warranted. This is even more apparent because the newly approved COVID-19 vaccine has been touted that the nanoparticles carrier could induce an allergic reaction in certain cases [97]. To determine the potential risk of silver and gold deposition in the human body, a long-term toxicity test is crucial to gather the safety data.

## CONCLUSION

The review describes the use of nanoparticles as non-alcohol-based antimicrobial agents. Though nanoparticle technology has some limitations, it provides an innovative approach to pharmaceutical technology. Nanoparticles offer a variety of advantages, such as enhancement of solubility and permeation, leading to better efficacy. In this article, we have reviewed some of the approaches commonly employed in improved antimicrobial therapy. Each of these approaches has its advantages and applications and provides a wider opportunity to pharmaceutical scientists to choose the right method as per the desired outcome. The use of metal nanoparticles is a common approach in developing an antimicrobial formulation. Gold and silver nanoparticles are the most researched metals in medicine and dentistry and have many possible applications. Besides gold and silver metallic nanoparticles, metal oxides of zinc, copper, and iron nanoparticles are also used extensively.

## ACKNOWLEDGEMENTS

### Conflict of interest

The authors report no conflict of interest.

### Authorship

KBL, AKJ, RS, SHK, FAR contributed to conceptualizing the study; KBL, AKJ, RS, SHK, FAR, LCM, AKh, AKa, PWN contributed to the search strategies ; KBL, LCM, AKh, AKa contributed to writing the original draft, LCM, AK, AK, PWN contributed to editing the manuscript, KBL, AKJ, RS, SHK, FAR, LCM, AK, AK, PWN contributed to data collection, KBL, AKJ, RS, SHK, FAR, LCM, AK, AK, PWN contributed to article review and data curation.
