# Supplementary material for: A review and revisit of nanoparticles for antimicrobial drug delivery
Source: J Med Life. 2022 Mar;15(3):328–35. doi: 10.25122/jml-2021-0097 (PMC9015166; doi:10.25122/jml-2021-0097)
Supplement: Supplemental data file. — Supplementary Table. References and characteristics of various nanoparticle applications. [file JMedLife-15-Supplementary.pdf]

Supplementary Table. References and characteristics of various nanoparticle applications.

S.No	Title	Drug delivery technology	Type of Nano system	Size	Nanomaterials used	Characteristics	*In-vitro* studies	*In-vivo* studies	Conclusion	Reference
1	Combination of Paclitaxel and R-flurbiprofen loaded PLGA nanoparticles suppresses glioblastoma growth on systemic administration	Chitosan modified poly-lactide-co-glycolic acid (PLGA) nanoparticles	Polymers coated Nanoparticles	200 nm	• Poly-lactic-co-glycolic acid<br>• Poly-lactic-co-glycolic acid<br>• Polyethylene glycol copolymer	• Particle size: 150–190 nm.<br>• Polydispersity index <0.2.<br>• Particle shape: spherical.<br>• Zeta potential: negative.<br>• Encapsulation efficiency: 80–95%.	Cytotoxicity and uptake characteristics using Rat glioma (RG2) cell line.	Implantation of RG2 cells in Wistar rats	PLGA NPs can efficiently carry their payloads to glioma tissue and potentiate the therapeutic activity	Toktas *et al.* (2020)
2	Investigation of ultrafine gold nanoparticles (AuNPs) based nano-formulation as single conjugates target delivery for improved methotrexate chemotherapy in breast cancer	Gold nanoparticles load Nano carrier drug delivery	Nano carrier	5 nm	• Hydrogen tetrachloroaurate (III) hydrate (HAuCl4)<br>• Sodium Borohydride (NaBH4, 98%, IR grade)<br>• Tri- sodium citrate (Na3C6H5O7·$2H_2O$, 99%, IR Grade)	• Percent yield: 86.4±2.2%<br>• MTX loading Efficiency: 78.8±2.7%	Cytotoxic efficacy on MCF7 cancer cell line using MTT viability assays.<br><br>The evaluation of cellular uptake was done by using HRTEM and ICP-AES.	Carried out in Ehrlich scites tumor (EAT) bearing Swiss albino mice<br><br>The concentration of AuNPs present in tumor tissues was determined by ICP-AES.<br><br>Histopathology examination of vital organs carried out to assess the efficacy and toxic effects	MTX conjugated AuNPs (AuNP-MTX) achieved significantly superior therapeutic efficacy over free MTX drug with no toxicity in in-vivo tumor mouse model.<br><br>Therefore, ultrafine AuNPs with better physicochemical properties exhibited potential to develop it as a nanocarrier for drug delivery for cancer treatment.	Naz *et al.* (2019)

Supplementary Table. Continued.

S.No	Title	Drug delivery technology	Type of Nano system	Size	Nanomaterials used	Characteristics	*In-vitro* studies	*In-vivo* studies	Conclusion	Reference
**3**	Lactoferrin/Hyaluronic acid double-coated lignosulfonate nanoparticles of quinacrine as a controlled release biodegradable nanomedicine targeting pancreatic cancer	Dual targeted quinacrine loaded lignosulfonate nanoparticles	Controlled release biodegradable nanomedicine	-	• Lignosulfonate	-	The in-vitro release profile was determined using dialysis method<br><br>Cellular cytotoxicity was assessed using MTT assay<br><br>Induction of apoptosis by QC solution or its nano-formulations was studied using Annexin-V assay by flow cytometry<br><br>Intracellular uptake of QC loaded formulations was examined capitalizing on the intrinsic fluorescence of QC	Anti-tumor activity was assessed in adult male BALB/c mice	The elaborated nanoparticles could be considered as a promising targeted nano-therapy for treatment of pancreatic cancer with higher efficacy & survival rate and lower organ toxicity.	Etman *et al.* (2020)
**4**	Magnetic nanocarriers for the specific delivery of siRNA: Contribution of breast cancer cells active targeting for down-regulation efficiency	Magnetic technology	Nano carrier	-	• Superparamagnetic iron oxide	• Particle size: 160 nm<br>• Zeta potential: +17 mV<br>• Polydispersity index: <0.3	BT-474 and SK-BR3 human breast carcinoma cell lines was used	-	TS-MSN are promising nanocarriers for the specific and efficient delivery of siRNA to HER2-overexpressing breast cancer cells.	Bruniaux *et al.* (2019)

Supplementary Table. Continued.

S.No	Title	Drug delivery technology	Type of Nano system	Size	Nanomaterials used	Characteristics	*In-vitro* studies	*In-vivo* studies	Conclusion	Reference
5	Development and evaluation of PLA-coated co-micellar nano-system of Resveratrol for the intra-articular treatment of arthritis	Polymeric based system	Mixed miscelles system	-	• Poloxamer 188<br>• Poloxamer 407<br>• Poly lactic acid	• Particle size: 52.97±4.52 nm Encapsulation<br>• Efficiency: 76.20±4.51<br>• Release efficiency: 76.26±6.25	*In-vitro* drug release was determined by the diffusion method using cellulose dialysis bags	Adult male Wistar rats was used to determine arthritis treatment	Intra-articular administration of the designed Resveratrol-loaded mixed micellar nano-system reduced the severity of cartilage lesions and synovial inflammation in the experimental arthritis model	Kamel *et al.* (2019)
6	Etoricoxib-loaded bio-adhesive hybridized polylactic acid-based nanoparticles as an intra-articular injection for the treatment of osteoarthritis	Polymeric nanoparticles	Bio-adhesive hybridized system	850 nm	• Polylactic acid<br>• (PLA)<br>• Pluronic L35 poloxamer 407<br>• And polyvinyl alcohol (PVA)	• Particle size: 884.450±150 nm versus 519.50±13.43 nm.<br>• PDI values: <0.7<br>• Zeta<br>• Potential: positive values<br>• Entrapment Efficiency: 91.05±2.19	*In-vitro* drug release was determined by the diffusion method using cellulose dialysis bags	-	Solvent technique is appropriate to formulate Etoricoxib-loaded bio-adhesive hybridized Nanoparticles<br><br>One formulation possessing the smallest particle size and the most sustained drug release showed promising results when examined for its ability to interact with the negatively charged sodium fluorescein.	Salama *et al.* (2020)

Supplementary Table. Continued.

S.No	Title	Drug delivery technology	Type of Nano system	Size	Nanomaterials used	Characteristics	*In-vitro* studies	*In-vivo* studies	Conclusion	Reference
7	Enhanced pulmonary delivery of fluticasone propionate in rodents by mucus-penetrating nanoparticles	Polymer based nanoparticles	Mucus penetrating Nano particles	200 nm	• Polylactide, grade 100DL7A<br>• Polylactide-co-poly (ethylene glycol), grade 100DL9K-mPEG2K	• Particle size: 200 nm<br>• Encapsulation efficiency: 30–35%	*In-vitro* release studies were performed using 0.5% w/v polysorbate 80 in 50 mM phosphate buffer (pH 7.4, ionic strength 0.13 M) as the release medium	Male rodents' lungs are used to determine the drug residence time	Pulmonary delivery of FP formulated as mucus penetrating nanoparticles achieved a higher local exposure in lungs of rodents when compared to free drug and even when compared to a mucoadhesive formulation with similar particle size	Popov *et al.* (2016)
8	Immune response elicited by an intranasally delivered HBsAg low-dose adsorbed to poly – caprolactone based nanoparticles	Polymeric based nanoparticles	Nasal vaccination	210 nm	• Poly-ε-caprolactone<br>• Chitosan	• Diameter: ≈208 nm<br>• low polydispersity (PI: 0.18)<br>• Zeta potential: +26 mV	-	Differentiated Caco-2 cells efficiently uptake PCL/chitosan NPs	The adjuvant ability of PCL/chitosan NPs as a nasal delivery system for HBsAg and the importance of the zeta potential of the formulation.<br><br>A dose-sparing activity of the formulations would therefore be beneficial in terms of a commercial approach of a nasal vaccine.	

Supplementary Table. Continued.

S.No	Title	Drug delivery technology	Type of Nano system	Size	Nanomaterials used	Characteristics	*In-vitro* studies	*In-vivo* studies	Conclusion	Reference
**9**	Influence of stabilizer type and concentration on the lung deposition and retention of resveratrol nanosuspension-in-microparticles	Polymer based system	Nano suspension micro particles	600 nm	• Mannitol<br>• Polyvinyl alcohol 205<br>• Sodium-dodecyl sulphate<br>• Alginate LFR5/60<br>• Chitosan hydrochloride<br>• Lactose	• Particle size: 604.9±23.4 nm<br>• Polydispersity index: 0.379±0.083<br>• Zeta potential: −21.0±0.98<br>• Drug content: 4.00±0.10	The aerodynamic performance of different formulations was investigated by a Next Generation Impactor (NGI, Copley Instruments Ltd, UK)<br><br>The *in-vitro* release of RES was investigated using paddle method equipped with USP dissolution apparatus	The residence time of different formulations in the lung, RES content in the Broncho alveolar lavage fluid (BALF) was analyzed at different time points after lung drug delivery in rats	The deposition and lung retention behavior of NS-in-MPs could be well tuned by selecting different type or concentration of stabilizers, which could facilitate local lung diseases therapy.	Liu *et al.* (2019)
**10**	Cationic nano-emulsions with prolonged retention time as promising carriers for ophthalmic delivery of tacrolimus	Polymer based drug delivery	Cationic Nano emulsion	-	• Poloxamer 188<br>• Kolliphor®<br>• Tween 80<br>• CKC<br>• Benzalkonium chloride	• Shape: Spherical<br>• Diameter: 178.8±2.7nm<br>• Zeta potential: +25.6±0.6 mV	Cytotoxicity study was conducted by human corneal epithelial cells (HCEC cells)	Gamma scintigraphy studies was carried out to determine precorneal residence time	Both in-vitro and in-vivo studies demonstrated safety and efficacy (ocular bioavailability of FK506).<br><br>The optimized formulation of FK506 CNE showed prolonged retention at the corneal surface.	Zhang *et al.* (2020)
**11**	Influence of different surfactants on the technological properties and *in-vivo* ocular tolerability of lipid nanoparticles	Lipid based system	Nanostructured lipid and polymeric systems.	-	• Dynasan1 114<br>• Kolliphor1 P188<br>• Kolliphor1 HS 15<br>• Cremophor1 A25<br>• Lipoid1 S100	-	-	Ocular tolerability test was done in Male New Zealand albino rabbits	The developed nano-particulate drug delivery system demonstrated excellent physio-chemical properties and found to be stable under the test conditions	Leonardi *et al.* (2014)

Supplementary Table. Continued.

S.No	Title	Drug delivery technology	Type of Nano system	Size	Nanomaterials used	Characteristics	*In-vitro* studies	*In-vivo* studies	Conclusion	Reference
**12**	Nanocomplexes based polyvinylpyrrolidone K-17PF for ocular drug delivery of naringenin	Polymer based system	Self-assembled Nanoparticles	-	• Polyvinyl pyrrolidone (K-17PF (17PF))	• Complexation efficiency: 98.51±0.86<br>• Diameter: 6.73±0.42 nm<br>• Polydispersity index: 0.254±0.019	Antioxidant activity and membrane permeation and cellular tolerance of NAR was assessed	Intraocular permeation of NAR and anti-inflammatory efficacy were assessed.	Nanocomplexes based on 17PF have great potential as novel nano-formulations to improve the ocular bioavailability and therapeutic efficacy of poorly water-soluble agents such as NAR.	Wang *et al.* (2020)
**13**	Novel ultrasmall nanomicelles based on rebaudioside A: A potential nanoplatform for the ocular delivery of pterostilbene	Lipid based system	Ultrasmall nanomicells	-	• Rebaudioside A	• Particle size: 3.99±0.03 nm<br>• Polydispersity index: 0.184±0.008	Antioxidant activity and membrane permeation was assessed.	The *in-vivo* studies were performed in experimental animals to determine the intraocular permeation and anti-inflammatory efficacy.	RA-based self-assembled ultrasmall nanomicelles demonstrate tremendous potential toward the improvement in the ocular bioavailability as well as improved therapeutic effect	Song *et al.* (2020)
**14**	Transcutaneous Anaesthetic Nano-enabled Hydrogels for Eyelid Surgery	Lipid based system	Self-nanoemulsifying drug delivery systems (SNEDDS)	300 nm	• Labrasol<br>• Capryol 90<br>• Transcutol P<br>• Carbopol 940	• Size: <300 nm<br>• Colloidal stability: Good<br>• Quasi spherical morphology<br>• Zeta Potential: -40.9±2.8	Franz cells diffusion studies	Ex vivo permeation studies were performed using tape stripping experiments	Nano-enabled anaesthetic hydrogels are suitable for non-invasive anaesthetic procedure for eyelid surgery. However elaborated studies are required	Lalatsa *et al.* (2019)

Supplementary Table. Continued.

S.No	Title	Drug delivery technology	Type of Nano system	Size	Nanomaterials used	Characteristics	*In-vitro* studies	*In-vivo* studies	Conclusion	Reference
**15**	Nano-lipoidal carriers of tretinoin with enhanced percutaneous absorption, photostability, biocompatibility and anti-psoriatic activity	Lipid based system	Liposomes, ethosomes, Solid lipid nanoparticles (SLNS) and nanostructured lipidic carriers (nlcs) Nano carriers	<200 nm	• Phosphatidylcholine<br>• Compritol 888 ATOGF3123<br>• Isopropyl myristate<br>• Butylated hydroxy toluene<br>• cholesterol	• Particle size: <200 nm. (182 nm (for liposomes) and 120 nm (for ethosomes), 82.3 nm (for SLNs) and 79.5 nm (for NLCs))<br>• Zeta-potential: liposomes (0.67 mV), ethosomes (−15.6 mV), SLNs (−20.1 mV) and NLCs (−23.5 mV)<br>• Entrapment efficiency: liposomes (65.01±2.31%), ethosomes (76.42±3.92%), SLNs (86.25±4.36%), NLCs (92.13±3.29%)	-	Skin permeation studies. Skin retention studies using mouse tail model	All the developed nanocarriers were found to be more biocompatible and effective than the marketed product. These findings could guide in proper selection of topical carriers.	Raza *et al.* (2013)

Supplementary Table. Continued.

S.No	Title	Drug delivery technology	Type of Nano system	Size	Nanomaterials used	Characteristics	*In-vitro* studies	*In-vivo* studies	Conclusion	Reference
**16**	Nanomachines and other caps on mesoporous silica nanoparticles for drug delivery	Nanomachines and mesoporous silica nanoparticles for drug delivery	Nanomachines	100 nm	• Mesoporous Silica	• Diameter: 100 nm<br>• Tubular pores: 2.5 nm with a total volume of about 1 cm^3/G	-	-	Mesoporous silica is a versatile platform for drug delivery Applications<br><br>The nanoparticles are nontoxic<br><br>The multifunctionality of nanotherapeutics including MSNs promises to revolutionize drug delivery.<br><br>Nanocarriers have great promise for therapeutic applications, and MSNs that can carry drugs to the site of a disease to produce a high local concentration without premature release and off-target damage may have the capability of realizing this goal.	Chen *et al.* (2019)

Supplementary Table. Continued.

S.No	Title	Drug delivery technology	Type of Nano system	Size	Nanomaterials used	Characteristics	*In-vitro* studies	*In-vivo* studies	Conclusion	Reference
**17**	Magnetic iron oxide nanoparticles as drug carriers: preparation, conjugation and delivery	Magnetic nanoparticles	The MNP carrier	10–100 nm	• Magnetic iron oxide nanoparticles	• Small size<br>• Facile chemical functionalization	-	-	Understanding of the preparation, conjugation and delivery processes will definitely bring, in the next decades, a novel magneto-nano-vehicle for effective theragnostic.	El-Boubbou (2018)
**18**	PEG-conjugated pyrrole-based polymers: One-pot multicomponent synthesis and self-assembly into soft nanoparticles for drug delivery	Soft Nanoparticles	-	<20 nm	• Polyethylene glycol grafted pyrrole-based conjugated polymers	• Size: 20 nm<br>• M.W.: 10.3 KdA	*In-vitro* glioblastoma multiforme as a model system was used with a PEGylated polypyrrole self-assembly system to deliver hydrophobic drugs to a cancer model and performed dose-response curves with free and encapsulated drugs	-	The developed system access to amphiphilic rod-coil PEGylated polypyrrole that self-assembles into soft nanoparticles thereby providing a high loading of hydrophobic drugs.	Moquin *et al.* (2019)

Supplementary Table. Continued.

S.No	Title	Drug delivery technology	Type of Nano system	Size	Nanomaterials used	Characteristics	*In-vitro* studies	*In-vivo* studies	Conclusion	Reference
**19**	Bone regeneration using bio-nanocomposite tissue reinforced with bioactive nanoparticles for femoral defect applications in medicine	Bioactive nanoparticles for femoral defect	Bio-nanocomposite	70–120 nm	• Chitosan (CS)–Hydroxyapatite (HA)–Wllastonite (WS) incorporated with zirconium	• Surface: Smooth<br>• Porosity:77–88%<br>• Biodegradable	Cyctotoxicity was assessed by MTT assay	-	The prepared scaffolds did not have cyto-toxicity at different concentrations of scaffold extracts. Consequently, the investigated scaffold can be beneficial in bone tissue engineering applications because of its simi-larity to natural bone structure and its proper porosity	Maghsoudlou *et al.* (2020)
**20**	Mesoporous silica nanoparticles as drug delivery vehicles in cancer	Nanoparticles as drug delivery	Mesoporous Silica NP	80 nm, 120 nm, 200 nm and 360 nm	• Silica Nanoparticle	• Size: 2–5 nm but larger pore sizes of 23 nm could also be generated	-	-	Mesoporous silica nanoparticles are a promising tool for innovative, more efficient and safer cancer therapy.	Watermann and Brieger (2017)
